# *Campylobacter jejuni pdxA* Affects Flagellum-Mediated Motility to Alter Host Colonization

**DOI:** 10.1371/journal.pone.0070418

**Published:** 2013-08-06

**Authors:** Hiroshi Asakura, Noritaka Hashii, Masashi Uema, Nana Kawasaki, Yoshiko Sugita-Konishi, Shizunobu Igimi, Shigeki Yamamoto

**Affiliations:** 1 Division of Biomedical Food Research, National Institute of Health Sciences, Setagaya-ku, Tokyo, Japan; 2 Division of Biological Chemistry and Biologicals, National Institute of Health Sciences, Setagaya-ku, Tokyo, Japan; 3 Department of Food and Life Sciences, Azabu University, Fuchinobe Chuo-ku, Sagamihara, Kanagawa, Japan; Charité-University Medicine Berlin, Germany

## Abstract

Vitamin B6 (pyridoxal-5'-phosphate, PLP) is linked to a variety of biological functions in prokaryotes. Here, we report that the *pdxA* (putative 4-hydroxy-L-threonine phosphate dehydrogenase) gene plays a pivotal role in the PLP-dependent regulation of flagellar motility, thereby altering host colonization in a leading foodborne pathogen, *Campylobacter jejuni*. A *C. jejuni pdxA* mutant failed to produce PLP and exhibited a coincident loss of flagellar motility. Mass spectrometric analyses showed a 3-fold reduction in the main flagellar glycan pseudaminic acid (Pse) associated with the disruption of *pdxA*. The *pdxA* mutant also exhibited reduced growth rates compared with the WT strain. Comparative metabolomic analyses revealed differences in respiratory/energy metabolism between WT *C. jejuni* and the *pdxA* mutant, providing a possible explanation for the differential growth fitness between the two strains. Consistent with the lack of flagellar motility, the *pdxA* mutant showed impaired motility-mediated responses (bacterial adhesion, ERK1/2 activation, and IL-8 production) in INT407 cells and reduced colonization of chickens compared with the WT strain. Overall, this study demonstrated that the *pdxA* gene affects the PLP-mediated flagellar motility function, mainly through alteration of Pse modification, and the disruption of this gene also alters the respiratory/energy metabolisms to potentially affect host colonization. Our data therefore present novel implications regarding the utility of PLP and its dependent enzymes as potent target(s) for the control of this pathogen in the poultry host.

## Introduction

*Campylobacter jejuni* is a Gram-negative, spiral-shaped, microaerophilic bacterium that causes foodborne diarrheal illness worldwide [1], [2]. Recent epidemiological and biochemical studies have shown that *Campylobacter* infection is also implicated in neuropathies, including Guillain-Barré syndrome (GBS), through the production of autoantibodies induced by bacterial lipooligosaccharides [3], [4]. The chicken is the predominant natural host for this pathogen, through which *Campylobacter* can be transmitted to humans [5], [6], [7]. Thus, control of this pathogen in poultry habitats is associated with the global public health benefit of preventing human campylobacteriosis. However, how to control *Campylobacter* remains unresolved, mainly due to our lack of understanding regarding how this pathogen colonizes chickens and establishes persistent infections and how it is involved in human virulence [7]. An increased understanding of the molecular biology of *Campylobacter* would therefore provide valuable information for the development of therapeutic strategies and vaccines targeting this pathogen.

The differential expression of metabolic gene products in relation to pathogenesis has largely been left unexplored. However, the role of gene regulation in this phenomenon is now receiving more attention, as the metabolism of bacterial pathogens may hold important clues for understanding their life cycles and host defense mechanisms [8], [9]. Vitamin B6 (pyridoxal-5’-phosphate, PLP) is an essential metabolic cofactor with numerous functions in more than one hundred enzymatic reactions in humans [10], [11]. Among prokaryotes, the biosynthesis of PLP has been intensively studied, mainly in *Escherichia coli,* revealing the involvement of two pathways with seven enzymatic steps [12], [13]. For a number of years, it was tacitly assumed that such pathways are ubiquitous in all organisms. However, the biological importance of vitamin B6 for bacterial pathogenesis has only recently been thoroughly investigated in other microorganisms, including *Mycobacterium tuberculosis* [14], *Bacillus subtilis* [15], and the *Campylobacter*-related microorganism *Helicobacter pylori* [16]. In *H. pylori*, a study involving a *pdxA* mutant recently demonstrated an essential role of the *pdxA* gene in flagellation, likely through inactivation of the flagellin glycosylation process (decoration with pseudaminic acid) [16]. There is no evidence regarding how the *pdxA* gene affects the process of flagellin glycosylation in *C. jejuni* similarly. However, a previous biochemical analysis showed that a UDP-4-keto-6-deoxy-GlnNAc aminotransferase (Cj1294) derived from *C. jejuni* generates UDP-4-amino-4,6-dideoxy-alNAc with the catalytic support of PLP as a co-factor under *in vitro* conditions [17], which led us to the assumption that PLP biosynthesis also affects flagellation and certain types of metabolism in this pathogen, thereby altering bacterial fitness and *in vivo* colonization, for which flagellar motility is a prerequisite [18].

Given this background, we studied the PLP biosynthesis pathway in *C. jejuni* through *in silico* prediction and mutagenesis analyses. Biochemical and phenotypic analyses showed that the lack of the *pdxA* gene abolished PLP production and impaired the ability of *C. jejuni* to form flagella. We then focused on this mutant to characterize its biological effect(s) on host colonization through biochemical, metabolomic, and host infection approaches.

## Materials and Methods

### Bacterial strains and media

The bacterial strains and plasmids used in this study are listed in Table 1. *C. jejuni* strain 81–176 [19] was grown using routine methods in Mueller-Hinton (MH) broth or on MH agar (Becton-Dickinson, Franklin Lakes, NJ, USA) at 37°C for 24 h in a humidified CO_2_ AnaeroPack-Microaero gas system (Mitsubishi Gas Chemicals, Tokyo, Japan). The media were supplemented with chloramphenicol (Cm) (20 μg ml^−1^) or kanamycin (Km) (30 μg ml^−1^), as appropriate. The *E. coli* DH5α strain, which was used as the host for subcloning and routine DNA manipulation, was grown in LB agar or LB broth unless otherwise indicated.

**Table 1:** Bacterial strains and plasmids used in this study.

Name	Description	Source/Reference
**Bacterial strain**		
WT	*C. jejuni* wild-type (WT) strain 81–176	[19]
pdxA−	*C. jejuni* 81–176 *pdxA* (CJJ81176_1253) mutant	This study
flaA−	*C. jejuni* 81–176 *flaA* (CJJ81176_1339) mutant	This study
pdxA-/+	pdxA- strain complemented with pRY-pdxA-Km	This study
DH5α	*E. coli* strain for DNA manipulation	Sigma-Aldrich
**Plasmid**		
pRY108, pRY109	Cm- or Km-resistant *C. jejuni*/*E. coli* shuttle vector	[21]
pGEM-pdxA-Cm	pGEM::*pdxA*-*cat* for homologous recombination	This study
pGEM-flaA-Cm	pGEM::*flaA*-*cat* for homologous recombination	This study
pRY-pdxA-Km	pRY109::*pdxAJ* for complementation	This study

### Construction of *C. jejuni* mutants and complementation of the *pdxA* mutant

The 81–176 mutant, in which most of the *pdxA* or *flaA* genes were replaced with a *cat* cassette (encoding a Cm-resistance protein), was constructed as described previously [20]. To construct a *pdxA* mutant, a 500-bp fragment upstream of the 5′ end and a 500-bp fragment downstream of the 3′ end of the *pdxA* locus were amplified from the wild-type (WT) strain *via* PCR using either pdxA-s and pdxA-as-BI or pdxA-s-BI and pdxA-as primers (Table S1). After *Bam*HI digestion, the two fragments were ligated and cloned into pGEM-T vector (Promega, Madison, WI, USA). A *cat* gene from the plasmid pRY109 [21] was then inserted into the *Bam*HI site in the pGEM-T plasmid, and this allelic exchange plasmid (pGEM-pdxA-Cm, Table 1) was introduced into the genome of strain 81–176 through natural transformation [22]. Successful transformants were selected on MH agar supplemented with 5% horse blood and Cm (20 μg ml^−1^). Allelic replacement was confirmed *via* nucleotide sequencing. Disruption of the *flaA* gene was performed in the same manner (the oligonucleotide primers used in these procedures are listed in Table S1). The *pdxAJ* locus and the upstream region predicted by the Neural Network Promoter Prediction program (http://www.fruitfly.org/seq_tools/promoter.html) to contain −35 and −10 promoter binding sites were amplified *via* PCR using the pdxA-CF and pdxA-CR primers (Table S1). The resultant PCR fragments were cloned into the *Xba*I/*Eco*RI sites of the pRY108 plasmid [21], yielding pRY-pdxA-Km (Table 1). This plasmid was introduced into the *pdxA* mutant strain through natural transformation, and the transformants were recovered on MH agar containing Km (10 µg ml^−1^) and Cm (20 µg ml^−1^). The construction of this pdxA−/+ mutant strain was confirmed *via* PCR using the pdxA-conF and pdxA-conR primers (Table S1).

### Quantification of PLP

Bacteria were grown microaerobically in 10 ml of MH broth to mid-logarithmic phase (an OD_600_ of 0.6), and crude homogenates were prepared in 20 mM Tris-HCl (pH 7.4) *via* bead crushing. After centrifugation for 10 min at 7,000 rpm at 4°C, the PLP contents in 50 µg of protein of the lysate and serial dilutions of the lysate were measured using a vitamin B6 ELISA kit (Uscn Life Science, Houston, TX, USA) according to the manufacturer’s instructions. Fresh MH broth was also tested for the measurement of PLP.

### Protein fractionation, SDS-PAGE, and immunoblotting

Membrane and cytoplasmic proteins from *C. jejuni* were isolated as described previously [23]. These protein samples were then separated on a 7.5% sodium dodecyl sulfate-polyacrylamide gel (SDS-PAGE) and stained with CBB (Coomassie Brilliant Blue) to visualize the protein profiles. The proteins on the gel were simultaneously transferred to a PVDF membrane (Merck-Millipore, Billerica, MA, USA), and the FlaA protein was detected using a rabbit polyclonal antibody generated against *C. jejuni* flagellin [24] and an HRP-conjugated goat anti-rabbit secondary antibody (GE Healthcare, Little Chalfont, UK). The blots were developed using the ECL detection system (GE Healthcare).

### Motility and growth assays

The motility of the WT, *pdxA* mutant, and complemented *C. jejuni* strains was assayed on 0.4% soft MH agar plates as previously described [25]. To measure bacterial growth, 1.4–1.9×10^6^ cells of *C. jejuni* that were microaerobically grown in MH broth to an OD_600_ of 1.2–1.3 at 37°C, were incubated in 10 ml of fresh MH broth supplemented with or without PLP (10 mg l^−1^) with agitation (120 rpm) for 0, 12, 24, 36, 48, 72 h. At each time points, turbidity of the medium was measured at 600 nm.

### Detection of pseudaminic acid (Pse)

#### (i) Derivatization of Pse

Pse was released from the 30 μg of cytosolic protein fractions from the WT and *pdxA* mutant strains grown in MH broth to OD_600_ of 0.55–0.60, using the GlycoProfile^TM^ β-elimination kit (Sigma-Aldrich) according to the manufacturer's instructions. The released Pse was labeled with 1,2-diamino-4,5- methylenedioxybenzene (DMB) using a sialic acid fluorescence-labeling kit (TaKaRa, Shiga, Japan), and the reaction mixture was applied to a solid-phase extraction cartridge (Envi-Carb C, Supelco, Bellefonte, PA, USA). After washing with 2.5 ml of 5 mM ammonium acetate (pH 9.6), the labeled Pse was eluted using 3 ml of 45% acetonitrile/5 mM ammonium acetate (pH 9.6) and freeze-dried. Fresh MH broth was also subjected to the above sample preparation to observe the effect of background growth medium.

#### (ii) Liquid chromatography/mass spectrometry (LC/MS)

Chromatographic separation of DMB-labeled Pse was performed using the Paradigm MS4 HPLC system (Michrom BioResource, Auburn, CA, USA). The separated DMB-labeled Pse was applied to a C18 trap column (L-column Micro, CERI) and eluted using 0.1% formic acid/2% acetonitrile (buffer A) and 0.1% formic acid/90% acetonitrile (buffer B) with a linear gradient of 10–90% buffer B over 30 min at a flow rate of 300 μl min^−1^. Mass spectrometric analysis of DMB-labeled Pse was performed using a Fourier transformation ion cyclotron resonance (FT-ICR)/ion trap (IT)-type mass spectrometer (LTQ-FT) (Thermo Electron, San Jose, CA, USA) equipped with a nanoelectrospray ion source (AMR, Tokyo, Japan). The presence of DMB-Pse was determined *via* sequential scans consisting of selected ion monitoring (SIM, *m*/*z* 441–461) using FT-ICR-MS and data-dependent MS/MS-MS/MS/MS/MS (MS^n^) with IT-MS.

### Detection of metabolic compounds

#### (i) Sample preparation

A total of 3.2–3.4×10^8^ *C. jejuni* WT or *pdxA* mutant cells grown in MH broth to an OD_600_ of 0.6 were trapped on a 0.4-μm filter membrane (Merck-Millipore), washed twice with 10 ml of water, and immersed in 2 ml of methanol containing 10 μM internal standard solution 1 (Human Metabolome Technologies (HMT), Yamagata, Japan). After sonication for 30 s, 1.6 ml of each suspension was mixed with 640 µL of water and 1.6 ml of chloroform, followed by centrifugation for 5 min at 2,300×*g*. The 750 µl of upper aqueous layer was filtered through a 5 KDa-cutoff filter (Millipore), lyophilized, and resuspended in 25 µl of water.

#### (ii) Capillary Electrophoresis-Time of Flight/mass spectrometry (CE-TOF/MS)

Cationic metabolites were analyzed using a fused silica capillary tube (50 μm×80 cm) and Cation Buffer Solution (HMT) in a capillary electrophoresis system equipped with a Time-of-Flight mass spectrometer (CE-TOF/MS) and a CE-ESI-MS sprayer (Agilent Technologies, Santa Clara, CA, USA). Electrospray ionization-mass spectrometry (ESI-MS) was conducted in positive ion mode at 4,000 V. Anionic metabolites were analyzed using a fused silica capillary and Anion Buffer Solution (HMT). ESI-MS was conducted in negative ion mode at 3,500 V. In both modes, the spectrometer was scanned from *m/z* 50 to 1,000. The other conditions were followed the cation analysis methodology of Soga and Heiger [26].

#### (iii) Data analysis

Raw data were processed using the MasterHands program [27]. Signal peaks corresponding to the isotopomers of 108 compounds (including the intermediates of the glycolytic system, the intermediates of the TCA cycle, and amino acids; see Table S2 for more details) were extracted. Each obtained migration time (MT) was normalized using the values of the internal standards. The resultant relative area values were further normalized based on the sample amounts. We used duplicate sets of samples from two independent experiments.

### ATP assay

To determine the intracellular ATP concentration of bacterial samples, the BacTiter-Glo Microbial Cell Viability assay kit (Promega) was used. After growing the bacteria in MH broth at 37°C under microaerobic conditions to an OD_595_ of 0.55–0.60, serial dilutions of all samples were prepared according to the manufacturer’s instructions. Following incubation of the samples at room temperature in a 96-well plate, luminescence was measured together with an ATP standard using GloMax Multi system (Promega), according to the manufacturer’s instructions. Simultaneously, we measured the bacterial numbers in the originally cultured MH broth by plate count.

### Cell adhesion assay, IL-8 measurements, and immunoblotting

INT407 cells were seeded into 24-well culture plates (TPP) (3.0×10^5^ cells well^−1^) and incubated in RPMI1640 medium (Life Technologies, Carlsbad, CA, USA) for 20 h at 37°C in a humidified CO_2_ incubator. The cells were then rinsed and inoculated with *C. jejuni* at a multiplicity of infection (m.o.i.) of 100. At 60 min post-infection, the cells were washed three times with PBS to remove non-adherent bacteria, followed by cell detachment using 0.1% saponin in PBS. Serial dilutions of the suspensions were plated onto MH agar to determine the numbers of viable, cell-associated bacteria. To measure IL-8 secretion from the INT407 cells after infection, INT407 cells were infected with *C. jejuni* at an m.o.i. of 100 for 0, 4, and 16 h, and the culture supernatants were used to measure IL-8 levels with a human IL-8 ELISA kit (Becton-Dickinson), according to the manufacturer’s instructions. ERK activation was examined *via* western blotting using tyrosine-phosphorylated and total ERK1/2 monoclonal antibodies (Cell Signaling Technology, Danvers, MA, USA).

### Chicken colonization assay

Specific pathogen-free, 14-day-old white leghorn chickens (obtained from Nisseiken Co., Ltd., Japan) were orally challenged with 500 μl of MH broth containing approximately 3.0×10^7^ WT or *pdxA C. jejuni* cells. The animals were euthanized at 7 and 28 days post-infection, and post-mortem cecal samples were collected after aseptic removal of the ceca. *C. jejuni* colonization of the cecum was examined by counting viable cells on mCCDA agar plates (Oxoid, Hampshire, UK). A control group was confirmed to be negative for *Campylobacter*. The above animal experiments were approved by the Committee for Animal Care and Use of the National Institute of Health Sciences, Japan.

### Statistics and web tool

The PATRIC prediction system (http://patricbrc.vbi.vt.edu/portal/portal/patric/Home), which assesses metabolic pathways in various prokaryotes based on their genomic sequences, was used to illustrate the putative PLP and Pse biosynthesis pathways in the *C. jejuni* 81–176 strain. The results from the motility, growth, ATP activity, cell adhesion, IL-8 production, and chicken colonization assays were expressed as the mean ± standard deviations of at least three independent observations. The significant differences between the measurements obtained from the WT and mutant strains were determined using Student’s *t*-test. *P* values <0.05 were considered statistically significant.

## Results

### Disruption of the *pdxA* gene abolishes PLP production in *C. jejuni*

**Figure 1:**
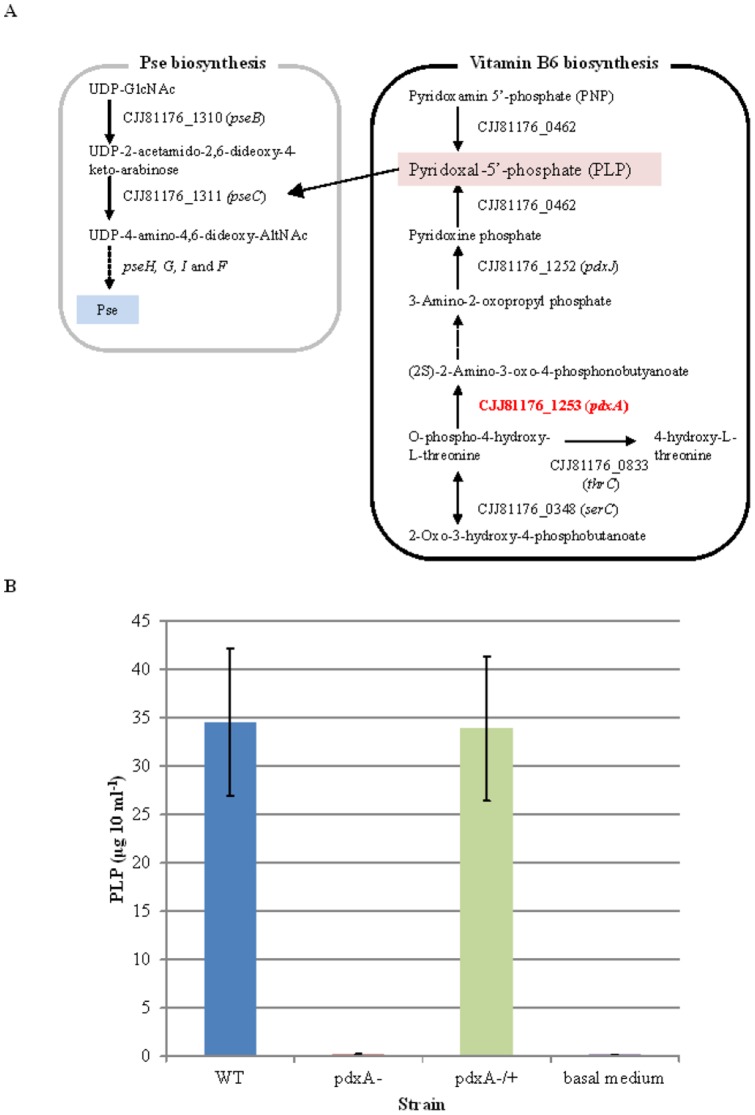
Inactivation of the *pdxA* gene impairs the biosynthesis of vitamin B6 (PLP) in *C. jejuni*. (A) A scheme for the PLP production pathway (right box) in *C. jejuni* in relation to Pse biosynthesis (left box) is illustrated based on *in silico* pathway analysis performed using PATRIC (http://patricbrc.vbi.vt.edu/portal/portal/patric/Home). (B) The *pdxA* mutant produced no PLP. The *C. jejuni* 81–176 WT, *pdxA* mutant, and the complemented strains were grown in 10ml of MH broth to an OD_600_ of 0.60. The suspensions were then homogenized, serially diluted, and subjected to ELISA to quantify the amounts of PLP (μg 10 ml^−1^). The data show the mean +/− standard deviations from three independent assays.

To predict the PLP biosynthesis pathway in *C. jejuni* 81–176, we used the *in silico* pathway tool PATRIC (http://www.patricbrc.org/portal/portal/patric/Home). The result of this prediction illustrated that at least five genes might constitute two independent pathways for PLP biosynthesis in this pathogen (right box, Fig. 1A). Among these genes, *pdxA* (CJJ81176_1253) and *pdxJ* (CJJ81176_1252) are known to be involved in the *de novo* synthesis of PLP in *E. coli* [12] and are, indeed, conserved in the *C. jejuni* genome [28]. Recently, Stahl and Stintzi [29] reported that the *pdxA* gene (Cj1239 in the NCTC11168 strain) may be essential for microbial growth. We therefore decided to use the *pdxA* gene to study the role of PLP biosynthesis in the biology of this pathogen and constructed an insertional *pdxA* mutant in *C. jejuni* strain 81–176. Biochemical assays collectively detected very less amounts of PLP (0.15±0.10 μg 10 ml^−1^) in the *pdxA* mutant than the WT strain (34.55±7.61 μg 10 ml^−1^), and complementation of the *pdxA* gene in the *pdxA* mutant restored PLP production (33.85±7.45 μg 10 ml^−1^) (Fig. 1B). Thus, we could demonstrate that the *pdxA* gene is truly a prerequisite for the PLP biosynthetic metabolism of this pathogen.

### The *pdxA* mutant impairs Pse production, flagellin glycosylation, and flagellation

**Figure 2:**
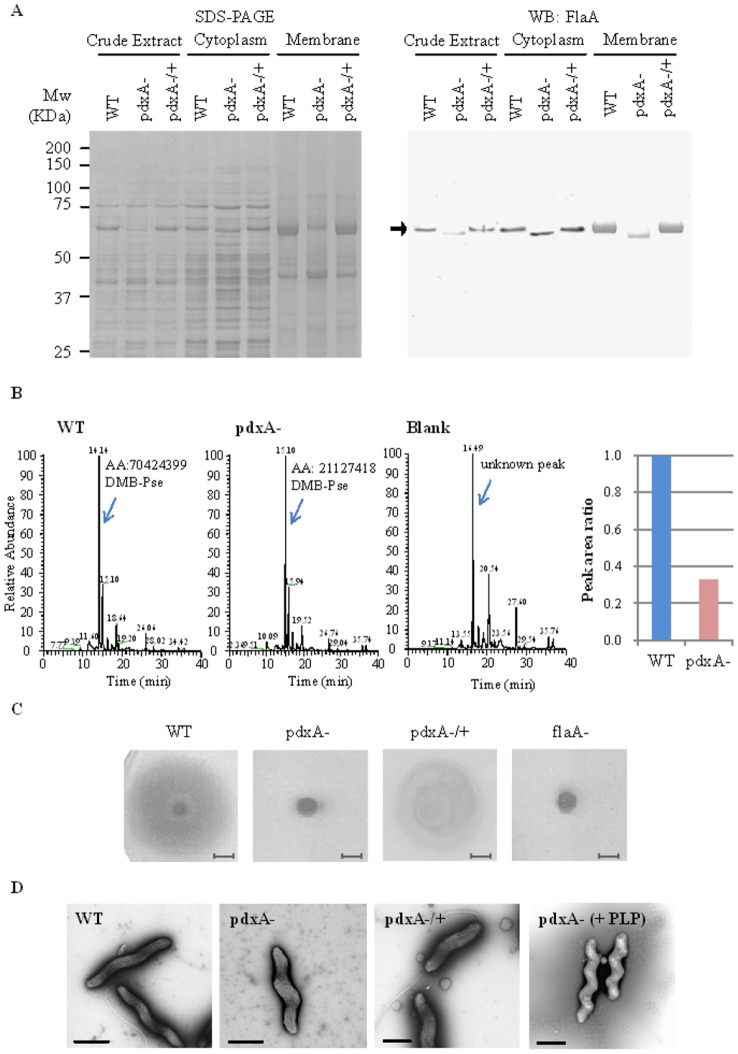
The *pdxA* mutant shows impaired pseudaminic acid (Pse) production and flagellar motility. (A) The *pdxA* mutant shows less glycosylation of FlaA. SDS-PAGE and western blotting were conducted to detect the *C. jejuni* FlaA protein. Crude extracts and subcellular (cytoplasmic and membrane) fractions were extracted from *C. jejuni* and visualized using CBB staining in an SDS-polyacrylamide gel (left panel). Western blot analyses were simultaneously performed to detect the FlaA protein (arrow, right panel). (B) The *pdxA* mutant shows reduced Pse production. The left panel shows an extracted ion chromatogram at *m*/*z* 441.0–461.0 obtained through SIM of DMB-labeled Pse from the WT and *pdxA* mutant strains (arrowed). The extracted ion chromatogram of blank sample (fresh MH broth) was simultaneously subjected to confirm the absence of Pse. AA, peak area in arbitrary units. Each ion signal is expressed as a relative percentage of the WT-derived sample (set to 100%) from two independent tests (right panel). MS^n^ data were shown in Fig. S1, S2, S3. (C) The disruption of the *pdxA* gene impairs motility of *C. jejuni*. The WT, *pdxA* mutant, *pdxA*-complemented (*pdxA*−/+), and *flaA* mutant (flaA-) strains were spotted and incubated onto 0.4% soft agar. Scale bars represent 3 mm. The motility of *pdxA* mutant was also assayed in the supplementation of 10 mg l^−1^ of PLP (pdxA− + PLP). (D) The *pdxA* mutant is aflagellated. Electron micrographs of the *C. jejuni* WT, *pdxA* mutant with or without supplementation of PLP (10 mg l^−1^), *pdxA*- complemented strains. The scale bars represent 1 μm.

*Campylobacter* flagellins are decorated with O-linked glycans, which are derivatives of Pse synthesized through sequential enzymatic reactions (i.e., transamination, decarboxylation, and racemization) [30], and this type of glycomodification is a prerequisite for the biogenesis, transport, and assembly of functional flagellar filaments [31], [32]. Among components of the Pse biosynthesis process, the *pseC* (Cj1294/CJJ81176_1311) gene product, UDP-4-keto-deoxy-GlcNAc transaminase, is reported to require PLP to generate UDP-4-amino-4,6-dideoxy-GalNAc, a spectrometric analintermediate in the synthesis of Pse (left box, Fig. 1A) [17]. Immunoblot analyses showed the less glycosylation but expression of the flagellin A (FlaA) in cytoplasmic fraction of the *pdxA* mutant compared with that of WT strain (Fig. 2A), and the complementation of the *pdxA* gene restored the glycosylation of FlaA in the *pdxA* mutant (Fig. 2A). Having less detection of FlaA in the membrane fraction of the *pdxA* mutant than that of the WT strain (Fig. 2A), it could be considered that the less glycosylated FlaA was not transported to the surface of the *pdxA* mutant. In agreement, mass spectrometric analyses clearly showed that the *pdxA* mutant produced approximately 3-fold less Pse than the WT strain (Fig. 2B, Fig. S1, S2, S3), providing a link between *pdxA*, PLP, and Pse biosynthesis in this pathogen. In consistent with the fact that the glycosylation and surface expression of flagellar filaments are prerequisite for bacterial motility [31], [32], phenotypic assays clearly demonstrated that the *pdxA* mutant was not motile, and the complementation of the *pdxA* gene restored motility (Fig. 2C). Furthermore, microscopic analyses consistently showed that the *pdxA* mutant did not generate any flagellar filaments and that complementation of the *pdxA* gene restored flagellation, likely to the same level as in the isogenic WT strain (Fig. 2D). Addition of PLP did not restore the flagellar production of the *pdxA* mutant (Fig. 2D). Together, we were able to demonstrate that disruption of the *pdxA* gene impaired the glycosylation of flagellin, thereby reducing bacterial motility.

### The *pdxA* mutant exhibits altered Pse biosynthetic metabolism

Considering that PLP mediates a variety of enzymatic processes [33], a comparative metabolomic analysis was performed to characterize/confirm the types of metabolism that might be related to PLP activity. CE-TOF/MS (capillary electrophoresis time-of-flight/mass spectrometry) analysis detected 99 metabolic compounds extracted from the WT and *pdxA* mutant strains, among which the levels of 10 and 6 compounds were either increased or reduced >2-fold, respectively, in the *pdxA* mutant compared with the WT strain (Table 2 and more detailed information in Table S2). In support of the link between the presence of the *pdxA* gene and Pse production, the *pdxA* mutant exhibited greater amounts of UDP-glucose (a Pse precursor, Fig. 1A left panel) and a PLP precursor, pyridoxamine-5’-phosphate (PNP, Fig. 1A right panel), which showed concentrations that were at 3.6-fold and 2.5-fold higher than were exhibited by the WT strain, respectively (Table 2). Thus, these data clearly indicated an essential role of the *pdxA* gene in Pse biosynthesis in *C. jejuni*.

**Table 2:** Representative metabolites that are altered between the *C. jejuni* WT and *pdxA* mutant strains.

Compound name	m/z ^*1^	MT ^*2^	Relative Area		Ratio ^*3^ (pdxA−/WT)
			WT	pdxA−	
***Increased***					
UDP-glucose/galactose	565.05	8.22	2.25E-04	7.99E-04	3.55
Azelaic acid	187.10	11.56	2.84E-04	9.31E-04	3.28
2-Amino-2-(hydroxymethyl)-1,3-propanediol	122.08	7.83	7.14E-04	2.01E-03	2.82
*cis*-Aconitic acid	173.01	26.16	3.36E-03	9.17E-03	2.73
ATP	505.99	11.16	4.37E-04	1.16E-03	2.66
GDP	442.02	10.11	1.91E-04	5.09E-04	2.66
Pyridoxamine-5'-phosphate (PNP)	249.06	9.78	2.25E-04	5.55E-04	2.46
β-Alanine	90.06	6.96	6.85E-04	1.65E-03	2.40
Glycine	76.04	7.87	4.58E-03	1.04E-02	2.27
Isocitrate	191.02	26.91	3.00E-03	6.39E-03	2.13
***Decreased***					
NADP^+^	742.07	8.92	5.3E-03	2.6E-03	0.49
ppGpp_divalent	300.47	13.69	4.3E-03	2.0E-03	0.48
Asparagine	133.06	9.80	1.3E-03	5.0E-04	0.40
Agmatine	131.13	4.94	5.5E-03	1.1E-03	0.19

The detected metabolites exhibiting >2.0-fold differences between the WT and pdxA- strains are shown. Each mean represents average from two independent tests. Candidate compounds are identified based on the detection peak (*m*/*z*)^*1^ and migration time (MT)^*2^ through HTM database. ^*3^ Relative mean of the pdxA-/WT ratio. Full lists are shown in Table S2.

### The *C. jejuni pdxA* mutant exhibits altered respiratory/energy metabolism

**Figure 3:**
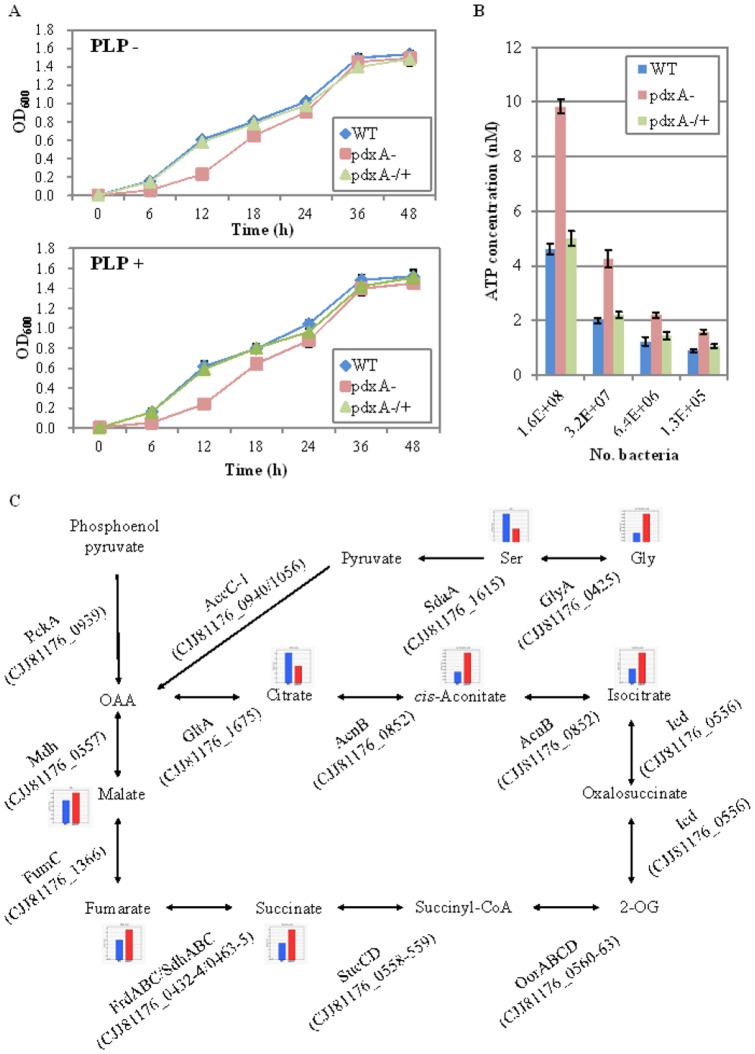
The *C. jejuni pdxA* mutant shows altered growth kinetics and respiratory/energy metabolism. (A) Growth curves of *C. jejuni* 81–176 WT, pdxA−, and the complemented mutant strains in MH broth not supplemented (left panel) or supplemented (right panel) with PLP (10 mg l^−1^). (B) Intracellular ATP levels of *C. jejuni* 81–176 WT, pdxA−, and the complemented mutant strains. ATP contents of four serial dilutions of the bacteria (shown as CFU 100 μl^−1^) under investigation were measured. The results are shown as means ± SD of data from triplicate wells of a representative experiment. (C) Focused dynamics of the *C. jejuni* TCA-cycle pathway. The pathway, the relative mean concentrations of the related metabolites in the WT (blue bars) and the *pdxA* mutant (red bars) strains, and the genes associated with the enzymatic conversion of each metabolite were illustrated with the PATRIC pathway analysis program.

As an additional characteristic, we notified that the *C. jejuni pdxA* mutant exhibited growth defect compared with the WT strain, but this mutant showed successive growth in the absence of PLP, indicating that the *pdxA* gene was not essential for the growth of this pathogen (Fig. 3A, left panel). Different from *H. pylori pdxA* mutant [16], the addition of PLP did not restore growth of the *C. jejuni pdxA* mutant (Fig. 3A). To investigate the metabolisms associated with the altered growth kinetics of the *C. jejuni pdxA* mutant, we thus focused indicators of significant alterations in energy/respiratory metabolisms because of the pivotal role of these metabolisms in the growth in this pathogen [34]. The metabolomic data showed that the *pdxA* mutant indeed produced greater amounts of ATP and GDP (by 2.7-fold each) and, hence, decreased amounts of NADP+ (0.5-fold) compared with the levels in the WT strain (Table 2). In agreement with these findings, the *pdxA* mutant exhibited an approximately 2.14-fold greater amount of ATP compared with the WT strain (when 1.6×10^8^ cells were assayed; Fig. 3B). Energy metabolism is well known to depend on the respiratory cycle. Corroborating this fact, the *pdxA* mutant showed alterations in the concentrations of TCA cycle intermediates including *cis*-aconitic acid (2.7-fold), isocitrate (2.1-fold), succinate (2.1-fold), malate (1.5-fold), citrate (0.6-fold), and serine (0.5-fold, a major carbon source for the respiratory cycle in this pathogen [35]) compared with the WT strain (Fig. 3C, Table 2, Table S2). Thus, we were able to show that the *C. jejuni pdxA* mutant exhibited altered growth and respiratory/energy metabolism.

### The *pdxA* mutant exhibits impaired *in vitro* cell adhesion and chicken colonization

**Figure 4:**
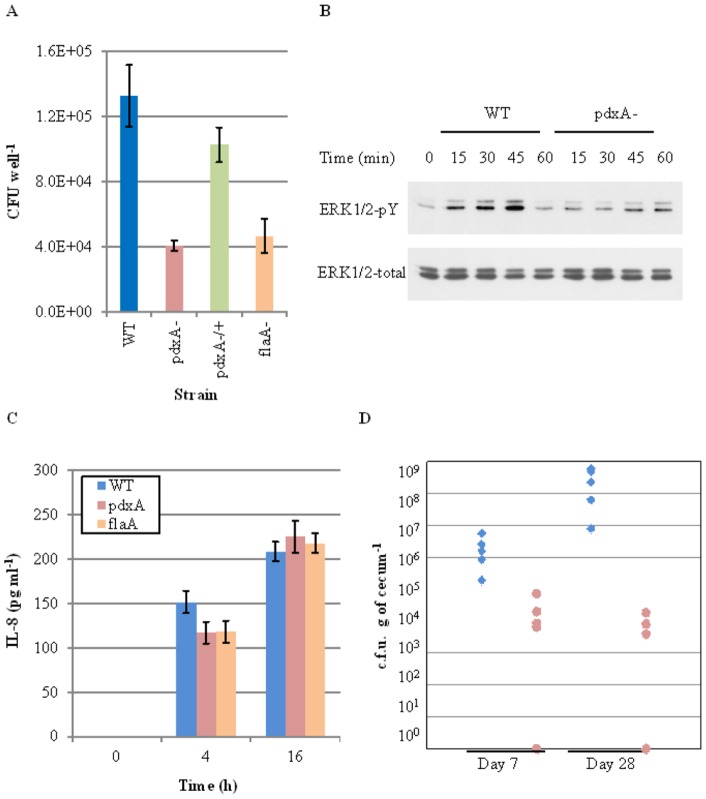
Deletion of the *pdxA* gene impairs *in vitro* cellular responses and *in vivo* colonization. (A) INT407 cells were infected for 1 h with the *C. jejuni* WT, pdxA−, pdxA−/+, and flaA− strains. The number of cell-adherent bacteria was measured by counting the plates after washing three times with PBS. (B) ERK1/2 activation upon infection. Western blotting was performed to detect the levels of phosphorylated and total ERK1/2 in the lysates from infected cells. (C) IL-8 production in INT407 cells was measured at 4 h and 16 h *p.i. via* ELISA. The data are presented in sections A and C as the mean values ± standard deviations from samples run in duplicate in at least three experiments. (D) Disruption of the *pdxA* gene reduces the colonization of the chicken cecum by *C. jejuni*. Groups of 14-day-old chickens (n = 10 per group) were orally inoculated with approximately 3×10^7^ CFU of WT or *pdxA* mutant *C. jejuni*. At 1 week and 4 weeks *p.i.*, the ceca were aseptically removed from the infected animals (n = 5 for each time point) and homogenized. Serial dilutions of the suspensions were plated on mCCDA agar to count CFU numbers. The closed diamonds and open circles represent the numbers of WT and *pdxA* mutant CFUs recovered from the animals, respectively.

*C. jejuni* requires flagellum-mediated motility for establishing the early phase of infection both *in vitro* [36] and *in vivo* [37], [38]. Accordingly, when INT407 cells were infected with the *pdxA* mutant, WT, and *flaA* mutant strains, the cell adhesion score of the *pdxA* mutant was found to be almost identical to that of the *flaA* mutant, exhibiting a 3-fold reduction compared with the WT strain at 1 h post infection (*p.i.*) (Fig. 4A). Following adherence, *C. jejuni* activates ERK1/2 MAPK signaling, which stimulates the production of interleukin (IL)-8 in INT407 cells [39]. In agreement with the above cell adhesion scores, the *pdxA* mutant caused delayed phosphorylation of ERK1/2 MAPK compared with the WT strain (Fig. 4B), which was similar to the *flaA* mutant [39]. Additionally, similar to the *flaA* mutant [39], the *pdxA* mutant induced significantly less IL-8 production in the INT407 cells compared with the WT strain at 4 h *p.i.* (*p*<0.05, Fig. 4C). A prolonged period after infection (18 h *p.i.*) resulted in normalization of the IL-8 secretion observed in response to all of the bacterial strains tested (Fig. 4C). These data indicated that the *pdxA* gene is a prerequisite for cell adhesion, with the mutant exhibiting delayed activation of ERK1/2 signaling and impaired IL-8 production in intestinal epithelial cells. We also tested the colonization ability of the *pdxA* mutant in chickens because this host represents the most important reservoir of the pathogen for human infection [5] as well as the fact that the flagellin mutants exhibited less colonization in chicken [38]. At 7 and 28 days after infection, the *pdxA* mutant exhibited approximately 100-fold (2.14±2.92×10^4^ CFU/g at day 7) and 4.6×10^4^-fold (6.00±7.48×10^3^ CFU/g at day 28) decreases in the colonization of chicken cecum tissues compared with the parental strain (2.14±2.12×10^6^ CFU/g at day 7 and 2.74±2.55×10^8^ CFU/g at day 28, both of which were significantly different (*p*<0.05) from the *pdxA* mutant-infected animals) (Fig. 4D). Together, we were able to demonstrate that disruption of the *pdxA* gene impaired the colonization of chicken intestine by *C. jejuni*.

## Discussion

Here, we examined the role of the PLP synthetic pathway in the biology of *C. jejuni.* Disruption of the *pdxA* gene clearly impaired PLP production. Mass spectrometric and biochemical analyses revealed a reduced production and glycosylation of flagellins in the *pdxA* mutant, which is likely to impair bacterial motility. Having the altered growth by disruption of the *pdxA* gene in this pathogen, we then performed comparative metabolomic approaches, further revealing the association of *pdxA* gene to energy/respiratory metabolisms. We finally showed that The *pdxA* mutant exhibited decreased cell adhesion-dependent responses *in vitro* and *in vivo* host colonization.

Based on the *in silico* pathway prediction for the PLP biogenesis, we selected the *pdxA* as a putative essential gene for the PLP production in this pathogen. A mutation of the *pdxA* gene impaired production of PLP in *C. jejuni* 81–176, supporting our prediction. The reduced Pse production in the *pdxA* mutant was also supportable to a previous study demonstrating the essentiality of PLP in the Pse production in *C. jejuni* [17]. The reduced production, but not complete loss of Pse in the *pdxA* mutant might be explained by the fact that small amounts of PLP (0.14±0.07 μg 10ml^−1^) were also detected from basal MH broth (Fig. 1B). Perhaps, the residual Pse peak in the *pdxA* mutant might be stem from residual PLP in the medium.

Since flagellin glycosylation is prerequisite for the biogenesis, transport, and assembly of flagellar filaments in this pathogen [31], [32] and which thereby alters the motility and host colonization of this pathogen [37], [38], it was plausible that the decreased Pse levels in the *pdxA* mutant, affected flagellar glycosylation, thereby altering transport of flagellin to the bacterial surface. Phenotypic and infection studies indeed showed impaired motility and host colonization of *C. jejuni* by disruption of the *pdxA* gene, supporting the idea that the reduced motility of the *pdxA* mutant was mainly due to the altered network between PLP and Pse.

We identified a link between PLP and the Pse modification system in *C. jejuni* 81–176, in agreement with the previously reported essential role of the *pdxA* gene in flagellar glycosylation in a closely related pathogen, *H. pylori* [16]. Moreover, the less glycosylation of FlaA protein in the cytoplasm of *pdxA* mutant was in agreement with the previous report demonstrating that the *C. jejuni pseC* mutant expressed unglycosylated FlaA in the cytoplasm [40]. Unlike *H. pylori*, however, the *C. jejuni pdxA* mutant could grow without supplementation of PLP, and the addition of PLP did not restore the motility and growth of *C. jejuni pdxA* mutant. These suggest the distinct metabolic impacts of PLP to the growth and/or viability between *H. pylori* and *C. jejuni.* A protein-protein network prediction tool, STRING database (http://string.embl.de/) indeed shows differential networks of the *pdxA* gene between the two pathogens (Fig. S4).

*Campylobacter* exhibits unique nutritional requirements, and it has been thought to only utilize amino acids and TCA cycle intermediates as carbon sources for energy production [41]. The TCA cycle is a sequential process involving enzymatic reactions in which a two-carbon acetyl unit is oxidized to CO_2_ and H_2_O to provide energy in the form of high-energy phosphate bonds. The different types of energy metabolism observed in the WT and *pdxA* mutant strains therefore suggested a possible link of PLP with these types of metabolism. Representative metabolites that were significantly altered by inactivation of the *pdxA* gene were thus discussed below.

### (i) Serine/Glycine

This pathogen exhibits a complete TCA cycle [42], and serine is particularly useful as a nutritional substrate that can be catabolized for growth and colonization in the chicken gut [37], [43], [44]. The decreased serine level detected would appear to be connected to glycine metabolism because *E. coli* serine hydroxymethyltransferase (GlyA) catalyzes the reversible interconversion of the amino acids serine and glycine using one-carbon tetrahydrofolate and PLP [45]. Thus, it could be considered that the imbalance between serine and glycine in the *pdxA* mutant might associate with the altered functionality of GlyA due to the lack of PLP.

### (ii) Citrate/*cis*-aconitate/isocitrate

These TCA intermediates are interconverted by aconitases [46], among which AcnB functions as the major TCA cycle enzyme in *E. coli* [47], [48]. Considering that *C. jejuni* 81–176 also harbors an *acnB* gene (CJJ81176_0852), the imbalance in these three TCA intermediates in the *pdxA* mutant might be due to reduced AcnB activity. AcnB forms an iron-sulfur cluster, thereby affecting its enzymatic activity [49]. Iron depletion has been shown to inhibit AcnB activity in *E. coli* [49], suggesting that the *pdxA* mutant might exhibit an altered iron metabolism and/or iron-sulfide cluster formation and, thus, reduced AcnB activity.

### (iii) Agmatin

Agmatin is a decarboxylation product of arginine that is involved in the urea cycle, the synthesis of creatine, and the generation of nitric oxide in eukaryotes [50]. The unaltered levels of arginine between the WT and *pdxA* mutant strains suggested that arginine decarboxylase (SpeA) might also require PLP for its activation. In support of this concept, *E. coli* SpeA shows a PLP-binding affinity [51], and a recent structural analysis showed that *C. jejuni* SpeA contains potent PLP-binding residues, similar to those of *E. coli* [52].

### (iv) β-alanine/asparagine

In contrast to the above three examples, the *pdxA* mutant exhibited an increased level of β-alanine, a precursor of coenzyme A (CoA), compared with the WT strain. β-alanine is mainly synthesized *via* the decarboxylation of L-aspartate in *E. coli* [53]. In this regard, the decreased levels of asparagine observed in the *pdxA* mutant suggested that asparaginase (AnsB), which is capable of deaminating periplasmic asparagine to aspartate [54], might be inactivated in this mutant, thereby causing the accumulation of asparagine, a precursor of β-alanine.

### (v) Glycolate

The *pdxA* mutant displayed decreased production of glycolate (hydroxylacetic acid), one of the smallest alpha-hydroxy acids (AHA). This metabolite is synthesized from 3-hydroxypyruvate (3HP) through reaction with glycoaldehyde, followed by decarboxylation, which requires PLP in *E. coli* [55], providing a possible reason for the decreased glycolate detected in the *pdxA* mutant.

Further studies will be necessary to elucidate the molecular impacts of PLP activity on the infection process in this pathogen through in-depth functional and/or structural analyses of each enzymatic reaction. Nevertheless, the data obtained in the present study provide the first evidence that biologically links PLP to the respiratory/energy metabolism as well as the flagellar glycosylation system, affecting the host colonization of *C. jejuni*.

It is likely that a number of factors could contribute to the colonization of chickens by *C. jejuni* (i.e., flagellum-mediated motility, chemotaxis, amino acid metabolism, energy metabolism, and iron utilization) [18]. The *in vivo* growth of *C. jejuni* has been argued to depend mainly on the availability of free amino and keto acids scavenged from the host or the intestinal microbiota [56]. The data reported herein therefore suggest that in addition to the decreased motility of the *pdxA* mutant, the altered levels of respiratory/energy metabolism might also participate in the impaired colonization of the chicken gut by this mutant. *In vivo* metabolic profiling of this pathogen would improve our understanding of the molecular basis underlying its adaptation to and interaction with the host and microbiota during infection.

In summary, this is the first report to demonstrate a functional role of the *pdxA* gene in altering the motility of and colonization of chickens by a leading foodborne pathogen, *Campylobacter jejuni*, including the demonstration of a novel link between PLP and flagellar glycosylation. PLP-dependent enzymes are likely to represent approximately 4% of the enzymes present in mammals [57], which attracted our interest in the investigation of PLP functions in terms of potential drug targets. Indeed, certain PLP-dependent enzymes are increasingly being identified as potential drug targets for the treatment of protozoan diseases [58], [59]. As poultry animals are the predominant reservoirs of this pathogen for human infection, our data reveal new prospects for potent targeting of PLP and its dependent enzymes to modulate the dynamics of and control this pathogen in livestock animals.

## Supporting Information

Figure S1 **Mass spectrum of DMB-labeled pseudaminic acid (Pse) acquired from the arrowed peaks in an extracted ion chromatogram at** ***m*** **/** ***z*** **441-2-461.2 obtained through SIM of DMB-labeled Pse from the** ***C. jejuni*** **81–176 wild type (WT),** ***pdxA*** **mutant (pdxA-), and fresh MH broth (blank) samples shown in** **Fig. 2B** **.**
(TIF)

Figure S2 **MS^n^ spectra of DMB-labeled pseudaminic acid (Pse) from the 81–176 wild type (WT).** (A) the MS/MS spectrum acquired from the molecular ion [M + H]+ (*m/z* 451.2) of peak (arrowed) in Fig. S1; (B) the MS/MS/MS spectrum acquired from the product ion (*m/z* 433.1) in the MS/MS; (C) the MS/MS/MS/MS spectrum acquired from the product ion (*m/z* 415.1) in the MS/MS/MS; (D) Fragmentation of DMB-labeled Pse. In addition to the DMB-labeled Pse, some ms/ms peaks were also detected. To indicate the molecular mass of these peaks, green ticks were used (to distinguish from the mass peaks).
(TIF)

Figure S3 **MS^n^ spectra of DMB-labeled pseudaminic acid (Pse) from the 81–176** ***pdxA*** **mutant.** (A) the MS/MS spectrum acquired from the molecular ion [M + H]+ (*m/z* 451.2) of peak (arrowed) in Fig. S1; (B) the MS/MS/MS spectrum acquired from the product ion (*m/z* 433.1) in the MS/MS; (C) the MS/MS/MS/MS spectrum acquired from the product ion (*m/z* 415.0) in the MS/MS/MS; (D) Fragmentation of DMB-labeled Pse. In addition to the DMB-labeled Pse, some ms/ms peaks were also detected. To indicate the molecular mass of these peaks, green ticks were used (to distinguish from the mass peaks).
(TIF)

Figure S4 **STRING network analysis.** Protein-protein network analysis was carried out using the STRING database (http://string.embl.de/). Protein entries from *C. jejuni* strain 81–176 or *H. pylori* strain G27 were used for the identification of putative protein-protein associations of PdxA to other bacterial proteins according to the guideline of the database.
(TIF)

Table S1 **Oligonucleotide primers used in this study.**
(XLSX)

Table S2 **Metabolic compounds in C. jejuni identified by CE-MS analysis.**
(XLS)
