# Supplementary figures and images for: Campylobacter jejuni pdxA Affects Flagellum-Mediated Motility to Alter Host Colonization
Source: PLoS One. 2013 Aug 6;8(8):e70418. doi: 10.1371/journal.pone.0070418 (PMC3735588; doi:10.1371/journal.pone.0070418)

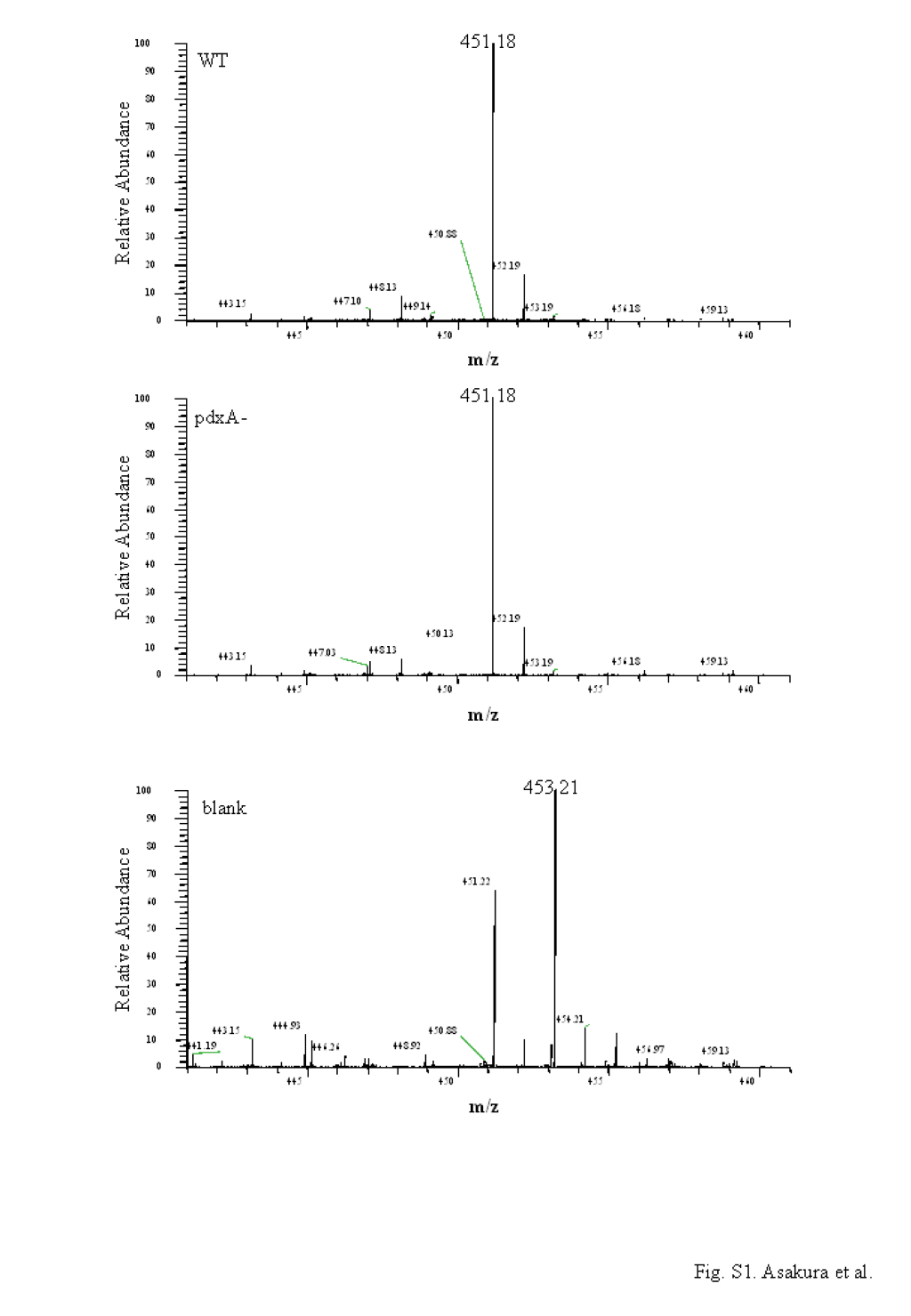

Supplement: Figure S1 — Mass spectrum of DMB-labeled pseudaminic acid (Pse) acquired from the arrowed peaks in an extracted ion chromatogram at m / z 441-2-461.2 obtained through SIM of DMB-labeled Pse from the C. jejuni 81–176 wild type (WT), pdxA mutant (pdxA-), and fresh MH broth (blank) samples shown in Fig. 2B . (TIF) [file pone.0070418.s001.tif]

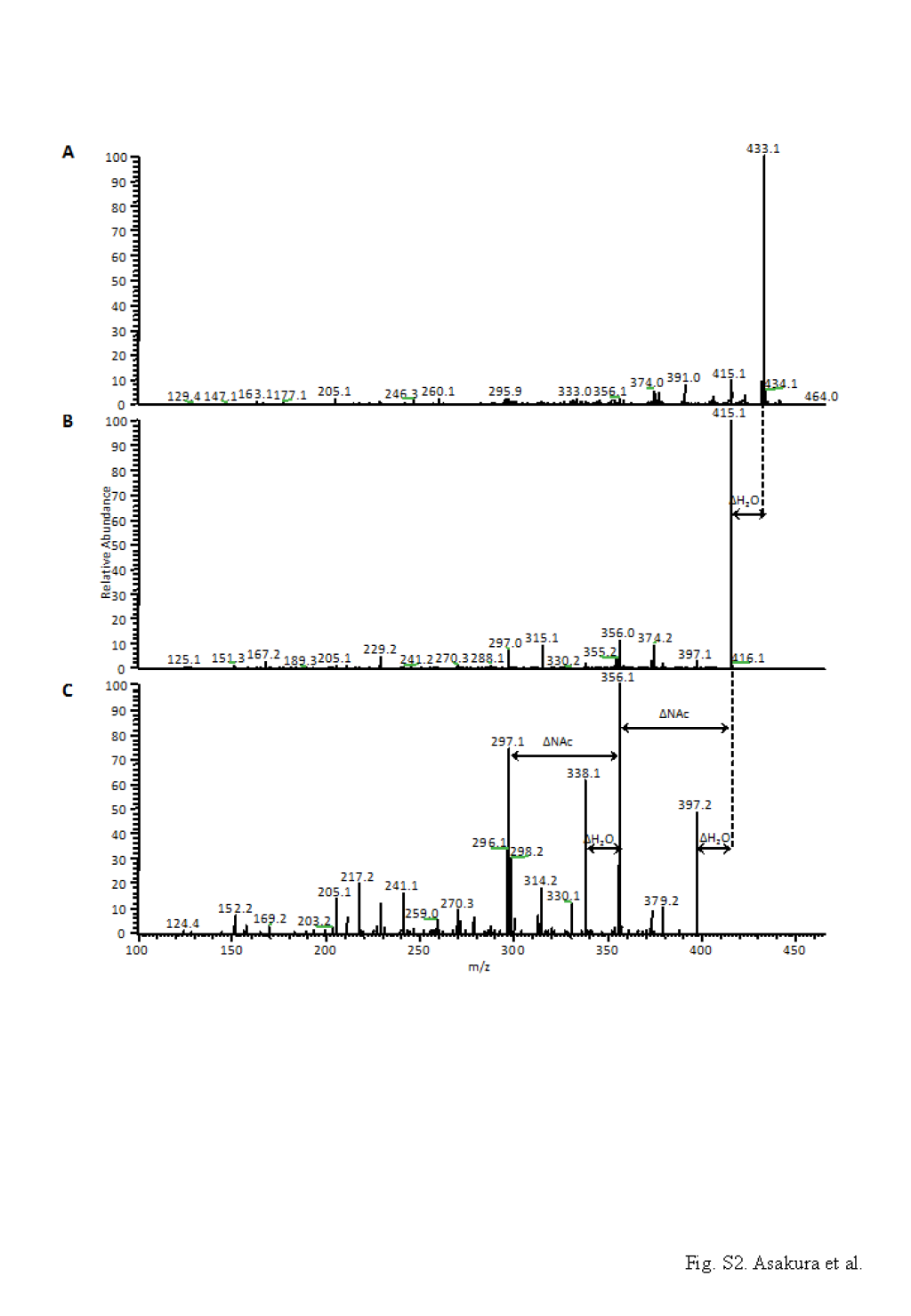

Supplement: Figure S2 — MSn spectra of DMB-labeled pseudaminic acid (Pse) from the 81–176 wild type (WT). (A) the MS/MS spectrum acquired from the molecular ion [M + H]+ (m/z 451.2) of peak (arrowed) in Fig. S1; (B) the MS/MS/MS spectrum acquired from the product ion (m/z 433.1) in the MS/MS; (C) the MS/MS/MS/MS spectrum acquired from the product ion (m/z 415.1) in the MS/MS/MS; (D) Fragmentation of DMB-labeled Pse. In addition to the DMB-labeled Pse, some ms/ms peaks were also detected. To indicate the molecular mass of these peaks, green ticks were used (to distinguish from the mass peaks). (TIF) [file pone.0070418.s002.tif]

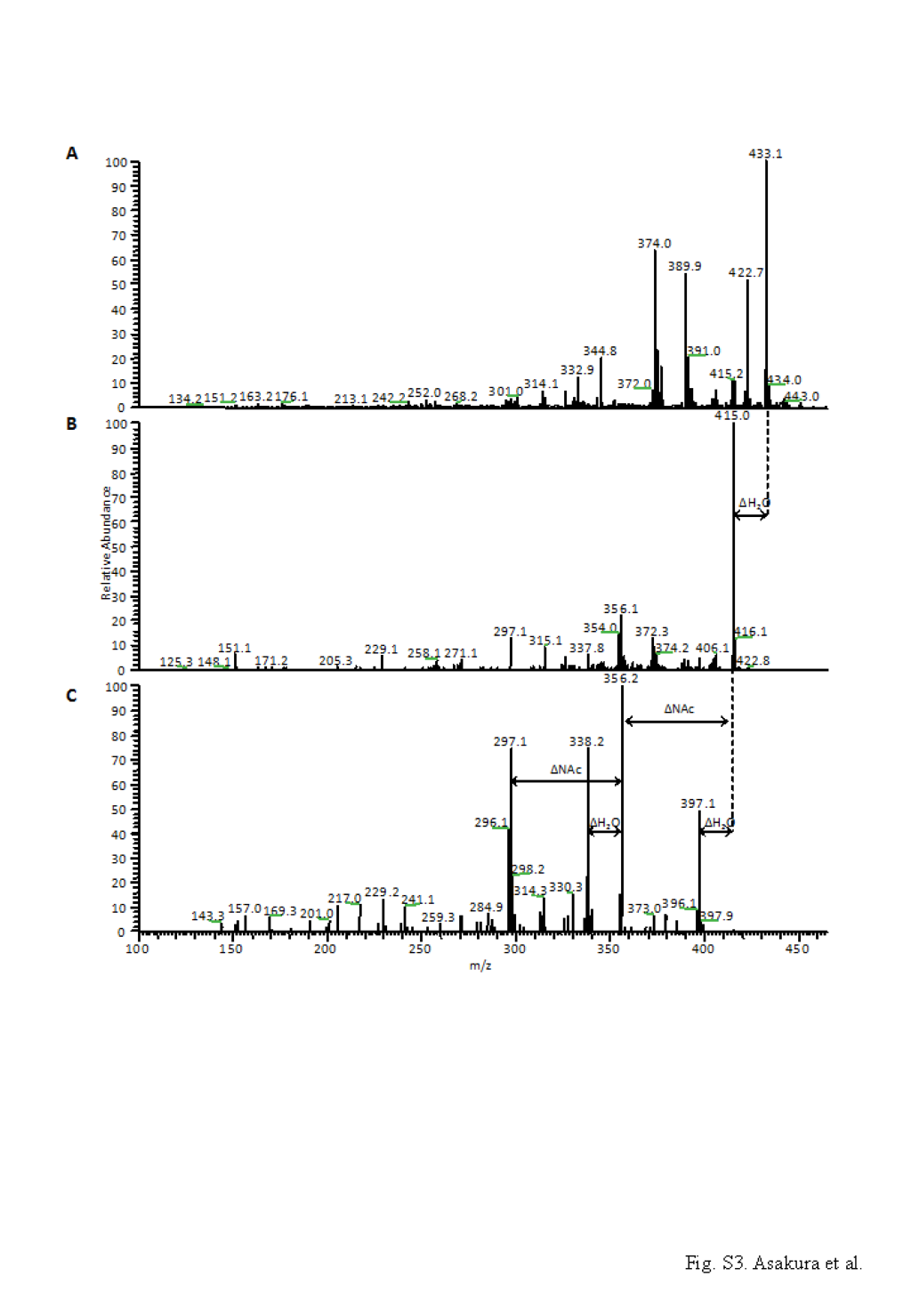

Supplement: Figure S3 — MSn spectra of DMB-labeled pseudaminic acid (Pse) from the 81–176 pdxA mutant. (A) the MS/MS spectrum acquired from the molecular ion [M + H]+ (m/z 451.2) of peak (arrowed) in Fig. S1; (B) the MS/MS/MS spectrum acquired from the product ion (m/z 433.1) in the MS/MS; (C) the MS/MS/MS/MS spectrum acquired from the product ion (m/z 415.0) in the MS/MS/MS; (D) Fragmentation of DMB-labeled Pse. In addition to the DMB-labeled Pse, some ms/ms peaks were also detected. To indicate the molecular mass of these peaks, green ticks were used (to distinguish from the mass peaks). (TIF) [file pone.0070418.s003.tif]

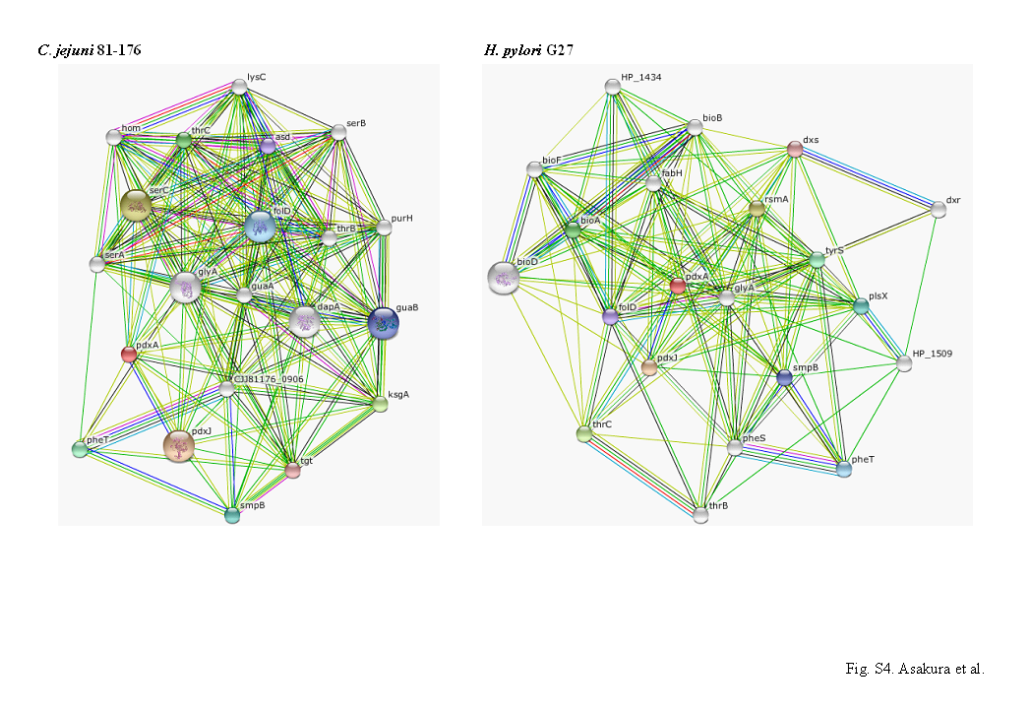

Supplement: Figure S4 — STRING network analysis. Protein-protein network analysis was carried out using the STRING database (http://string.embl.de/). Protein entries from C. jejuni strain 81–176 or H. pylori strain G27 were used for the identification of putative protein-protein associations of PdxA to other bacterial proteins according to the guideline of the database. (TIF) [file pone.0070418.s004.tif]
